# Human Beta Casein Fragment (54–59) Modulates *M. bovis* BCG Survival and Basic Transcription Factor 3 (BTF3) Expression in THP-1 Cell Line

**DOI:** 10.1371/journal.pone.0045905

**Published:** 2012-09-28

**Authors:** Dharamsheela Thakur, Reshu Saxena, Vandana Singh, Wahajul Haq, S. B. Katti, Bhupendra Narain Singh, Raj Kamal Tripathi

**Affiliations:** 1 Division of Toxicology, Central Drug Research Institute, Lucknow, India; 2 Division of Microbiology, Central Drug Research Institute, Lucknow, India; 3 Division of Medicinal and Process Chemistry, Central Drug Research Institute, Lucknow, India; Sudbury Regional Hospital, Canada

## Abstract

Immunostimulatory peptides potentiate the immune system of the host and are being used as a viable adjunct to established therapeutic modalities in treatment of cancer and microbial infections. Several peptides derived from milk protein have been reported to induce immunostimulatory activity. Human β -casein fragment (54–59), natural sequence peptide (NS) carrying the Val-Glu-Pro-Ile-Pro-Tyr amino acid residues, was reported to activate the macrophages and impart potent immunostimulatory activity. In present study, we found that this peptide increases the clearance of *M. bovis* BCG from THP-1 cell line in vitro. The key biomolecules, involved in the clearance of BCG from macrophage like, nitric oxide, pro-inflammatory cytokines and chemokines, were not found to be significantly altered after peptide treatment in comparison to the untreated control. Using proteomic approach we found that BTF3a, an isoform of the Basic Transcription Factor, BTF3, was down regulated in THP-1 cell line after peptide treatment. This was reconfirmed by real time RT-PCR and western blotting. We report the BTF3a as a novel target of this hexapeptide. Based on the earlier findings and the results from the present studies, we suggest that the down regulation of BTF3a following the peptide treatment may augment the *M. bovis* BCG mediated apoptosis resulting in enhanced clearance of *M. bovis* BCG from THP-1 cell line.

## Introduction

Peptide therapy is being increasingly used in clinical applications and certain naturally derived peptides have been successfully used for many years [1]. The successful usage of many peptides with known immunostimulatory properties as a viable adjunct to established therapeutic modalities allows the emergence of a novel approach for the treatment of infectious and malignant conditions in coming decades. Since the discovery of muramyl dipeptide (MDP), the smallest fragment of bacterial peptidoglycan carrying immunostimulatory activity in 1974 [2], [3], [4], efforts were on to develop chemically defined low molecular weight substances as immunostimulating agents. Consequently, a number of highly potent compounds have been identified and some of these are reported to be under clinical trials at present. Novel chemically well-defined and clinically acceptable immune modulators and several glycopeptides and lipopeptides with close structural resemblance to MDP have been designed and synthesized. However, most of them have microbial origin and are therefore associated with some toxic side effects.

An immunostimulatory hexapeptide corresponding to fragment 54–59 of human β-casein, carrying Val-Glu-Pro-Ile-Pro-tyr amino acid residues, has been reported to stimulate the phagocytosis of opsonized sheep red cells by murine peritoneal macrophages in vitro and to enhance the resistance of adult mice to infection with *Klebsiella pneumoniae* following intravenous administration [5]. This peptide has also been reported to increase the nitric oxide release from neutrophils [6] and to reduce *Leishmania donovani* burden in host when used prophylactically by stimulating the host’s resistance to parasite [7]. Being a food protein derivative this peptide is likely to be devoid of undesired side effects associated with the substances of microbial origin. Thus, in present study we examined the effect of this peptide on macrophage activation and subsequently on the survival of recombinant *Mycobacterium bovis* BCG inside THP-1 cell line. In order to elucidate the probable molecular mechanisms for the reducing number of bacilli inside the THP-1 cell, different markers of macrophage activation like, nitric oxide, pro-inflammatory cytokine and chemokines were analysed. Proteome analysis of the THP-1 cell line after treatment with this peptide was performed to identify the candidate proteins responsible for the reduced survival of the mycobacterium. Treatment with this peptide decreased the survival of *M. bovis* BCG inside the THP-1 cell, and caused the down regulation of BTF3a, an isoform of the Basic transcription factor, BTF3.

## Results

### Beta Casein Fragment (54–59) Decreases the Survival of *M. bovis* BCG in the THP-1 Cell Line

**Figure 1:**
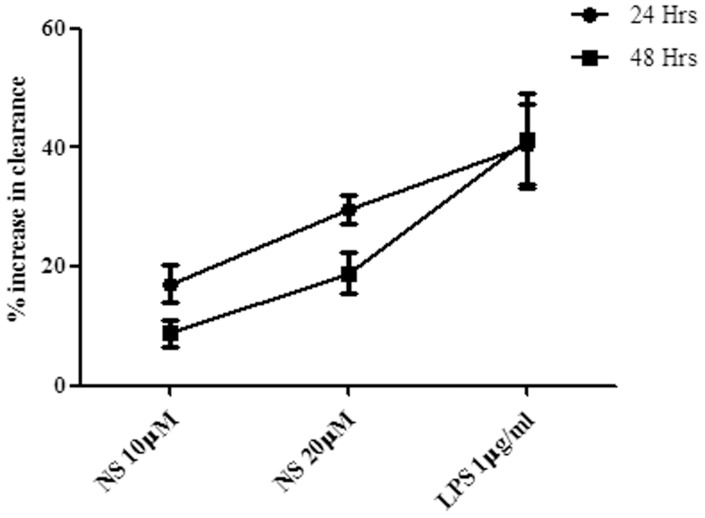
Clearance of *M. bovis* BCG from THP-1. PMA differentiated THP-1 cells were treated with NS at 10 µM and 20 µM and infected with bioluminescent recombinant BCG. RLU was measured at 0, 24 and 48 hrs time point of infection. The percent decrease in RLU reading after 24 hrs and 48 hrs of infection was considered as percent clearance of bacilli from THP-1 over this time period of infection. Percent increase in clearance in treated cells was calculated in reference to control cells. Lipopolysaccharide (LPS) was taken as positive control for the study. The values and error bars represent average and standard deviations of three independent set of experiments.

We tested the efficacy of Beta casein fragment (54–59) natural sequence peptide (NS) on the clearance of *M. bovis* BCG from THP-1 cells. Recombinant BCG showing constitutive luciferase expression was used for the study. Change in RLU value was recorded after different time point of infection and percent increase in clearance in treated cells with respect to control cells was calculated. Treatment with this peptide enhanced the clearance of *M. bovis* BCG over untreated control cell in a dose dependent manner. Cells treated with 10 µM and 20 µM peptide showed 17.13% and 29.56% increase in clearance of BCG, respectively after 24 hours of infection as compared to untreated control (Fig. 1). The percent increase in clearance was less after 48 hrs of infection as compared to 24 hrs time period; only 8.75% and 18.93% increase in clearance was obtained upon treatment with 10 µM and 20 µM peptide, respectively. Treatment with *E. coli* lipopolysaccharide (LPS, 1 µg/ml), a known macrophage activator, enhanced the clearance of BCG by 40.53% and 41.22% as compared to untreated control after 24 and 48 hrs of infection, respectively.

### Nitric Oxide Production from THP-1 Cell Line

**Figure 2:**
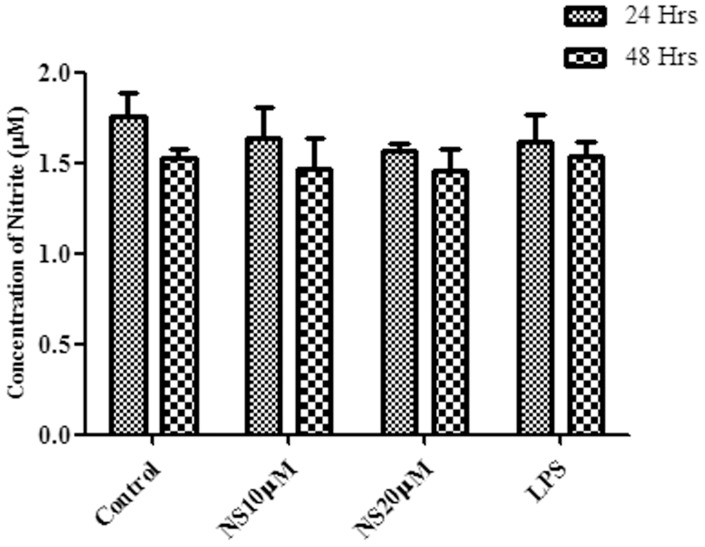
Nitric oxide production from THP-1 after *M. bovis* BCG infection. PMA differentiated THP-1 cells pretreated with NS at 10 µM and 20 µM were infected with recombinant BCG. Infection was continued in the presence of peptide for 24 and 48 hrs. Nitric oxide produced in cell culture after 24 and 48 hrs of infection was measured as described in materials and methods. Lipopolysaccharide (LPS) was taken as a positive control. The values and error bars represent average and standard deviations of three independent set of experiments. Student T test was performed to find out significant difference between control and treated group at P<0.01–0.05.

Nitric oxide (NO) content in the cell supernatant was calculated after 24 and 48 hours of infection in control and NS treated cells. NO level was found to be similar in control, NS and LPS treated cells after 24 and 48 hrs of infection (Fig. 2).

### Real Time PCR Analysis of Pro-inflammatory Cytokine and Chemokine Production

**Figure 3:**
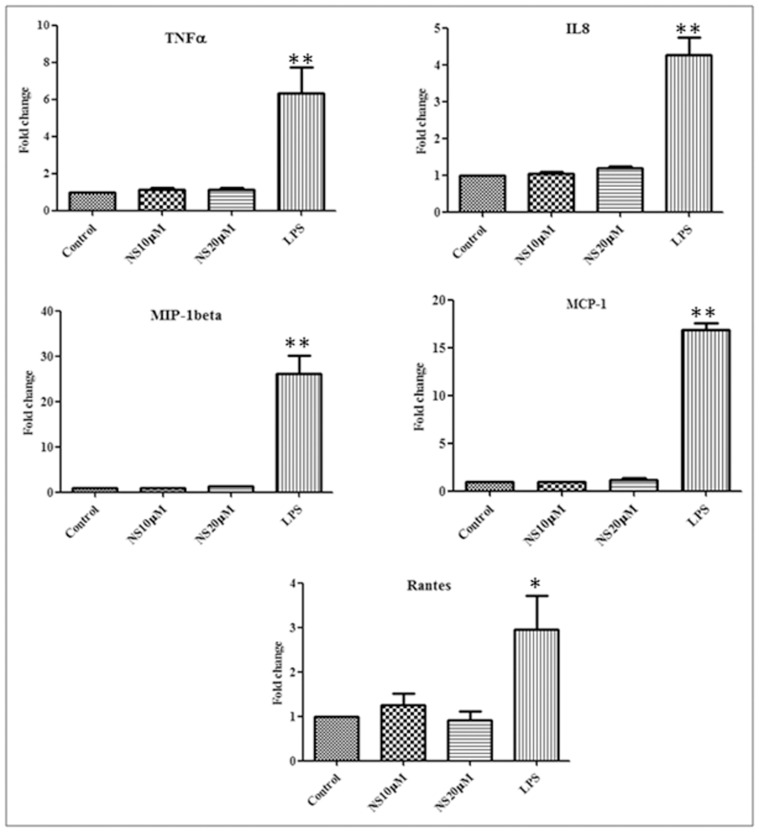
Change in pro-inflammatory cytokine and Chemokine expression from THP-1 upon peptide treatment. PMA differentiated THP-1 cells were treated with NS at 10 µM and 20 µM, Lipopolysaccharide (LPS) at 1 µg/ml concentration for 6 hrs. Change in TNF-α, IL-8, MIP-1beta, MCP-1 & Rantes expression level was quantified by real-time RT-PCR and expressed as fold change in expression of cytokines and cytokines in treated cells verses control cells. LPS was taken as a positive control. Bar shows the mean fold change ± SEM from three different experiments. The difference in cytokine expression between experimental group and control group was assessed by Student’s t-test and comparisons were considered significantly different at P≤0.05 (*) and at P<0.01 (**).

Real time PCR was performed to examine the effects of NS on the expression of pro-inflammatory cytokine (TNF-α) and chemokines (IL-8, MCP-1, MIP-1β and Rantes) at transcriptional level. No significant change was noticed in the transcripts level of pro-inflammatory cytokine and chemokines in NS cells, treated at 10 µM and 20 µM in comparison to control. However, treatment with LPS (1 µg/ml) caused a significant change in the expression of all these cytokines and chemokines as compared to the untreated control (Fig. 3).

### Densitometric Analysis of Differentially Expressed Protein Spots

**Figure 4:**
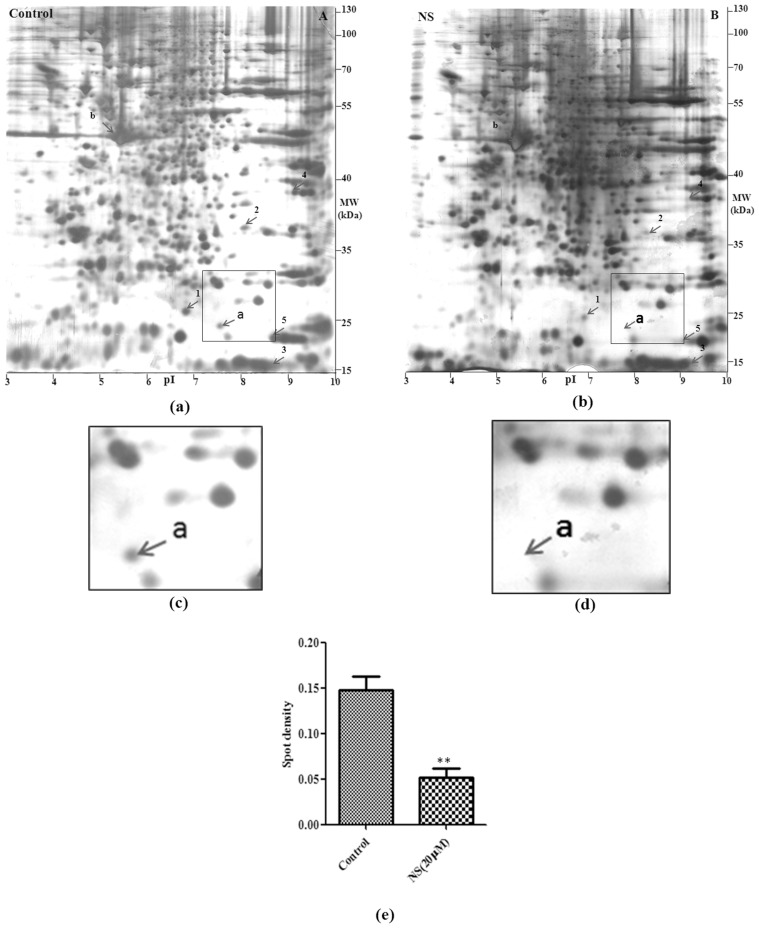
Proteome profile of THP-1 cells after NS treatment. Proteome profile of control untreated sample (panel- a) and NS treated sample (panel-b) are shown. The pI scale was constructed based on the dimensions of the linear pH gradient strip (pH range 3–10 linear over 13 cm). Protein spot **a** was found to be down regulated in NS treated sample (enlarged panel). Mean density of protein spot a in control and NS treated samples is shown in panel-e. Results are expressed as mean ± SEM, *n* = 3. The difference in protein spot density between treated and control group was assessed by Student’s t-test and comparisons were considered significantly different at P<0.01 (**).

The density of each spot was calculated using Image Master 2D Platinum software (GE Healthcare). Comparison of spot densities from three experiments showed that NS treatment at 20 µM concentration significantly reduced the expression of a protein spot no. a (*P*<0.01 by ANOVA) (Fig. 4 a&b). This spot was found to be down regulated by 2.84 fold in treated sample as compared to untreated control (Fig. 4e). Five additional proteins spots (spot no. 1–5) showed differences in expression level, ranging from 2.3 fold to nearly 10 fold, but were statistically not significant. This lack of statistical significance was due to large variations in the densities of these spots among the three independent experiments. Spot b labelled in gel picture corresponds to actin protein (MW- 42 kDa, pI - 5.3) and was considered as one of the marker protein for normalization.

### Identification of BTF3 Protein

**Figure 5:**
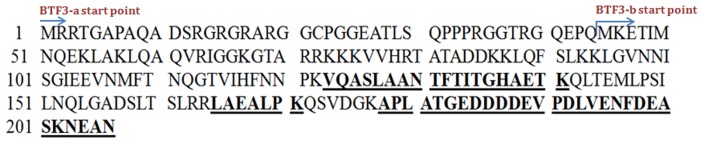
Sequence of BTF3 protein. Peptides which matched with BTF3 protein sequence after sequencing of spot (a) by LC-MS/MS are shown as bold underlined. BTF3 has two isoforms BTF3a and BTF3b. The BTF3a carries additional 44 amino acid residues at its N terminus which is absent in BTF3b. Matched peptides are common to BTF3a and BTF3b isoforms.

Protein spot a, showing differential expression was digested by trypsin and peptides were analysed by electrospray ionisation mass spectrometry. Three peptides corresponding to 123–141 (VQASLAANTFTITGHAETK), 165–171 (LAEALPK) and 178–206 (APLATGEDDD DEVPDLVENFDEASKNEAN) of the Basic Transcription Factor (BTF3) were identified. Total sequence coverage of the matched peptides was 26% of the total BTF3 protein sequence as shown in Fig. 5. Matched peptides were present towards C terminal end of BTF3 and this region was common in both BTF3a and BTF3b isoforms (Fig. 5).

### Differential Expression of BTF3 Isoforms

**Figure 6:**
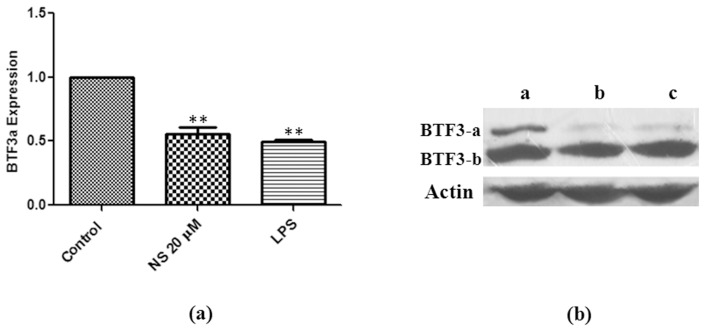
BTF3a is down regulated after NS treatment. Real Time RT-PCR (a) and western blotting (b) was performed to analyse the expression of BTF3. BTF3a expression was down regulated after NS and LPS treatment. Fold change in expression during treated conditions is presented in respect to control. The values and error bars represent average and standard deviations of three independent set of experiments. Student T test was performed to find out significant difference between control and treated conditions and comparisons were considered significantly different at P<0.01 (**). In western, two different isoforms of BTF3 were detected as shown in figure (Lanes: a - control, b - NS-20 µM, c - LPS-1 µg/ml). Lower panel depicts the equal level of actin protein which was taken as loading control.

Real Time RT-PCR was performed to examine the differential expression of BTF3 isoforms at transcript level. BTF3a isoform was down regulated by 1.8 fold and 2 fold on treatment with NS (20 µM) and LPS (1 µg/ml), respectively (Fig. 6a). Since BTF3b sequences were common with BTF3a we could not examine BTF3b specific expression by real time PCR. Instead, we performed western blotting using anti-BTF3 antibody in order to discern the expression levels of BTF3 isoforms. Two isoforms of BTF3, BTF3a and BTF3b corresponding to 23 and 18 KDa, respectively were detected. BTF3b isoform was found to be the predominant. A significant down regulation of BTF3a was noticed after treatment with NS at 20 µM concentration, whereas no appreciable change was observed in the expression of BTF3b. LPS treatment caused similar down regulation of BTF3a isoform without altering the expression of BTF3b (Fig. 6b).

## Discussion

Beta casein fragment (54–59) natural sequence peptide (NS) was reported to exhibit immunostimulatory activity. This peptide increased the phagocytosis and enhanced the resistance of adult mice to infection with *Klebsiella pneumoniae* following intravenous administration [5], and reduced *Leishmania donovani* burden in host when used prophylactically by stimulating the host’s resistance to parasite [7]. In present study, we found that the NS peptide decreased the survival of *M. bovis* BCG in THP-1 cell line in a dose dependent manner. LPS, a known macrophage activator, also showed enhanced clearance of the bacilli from THP-1 cells. Since NS peptide reportedly exert it`s immunostimulatory activity through macrophage activation, the peptide mediated macrophage activation may lead to increased clearance of *M. bovis* BCG, as tubercle bacilli reside mainly within host macrophages and the activation of macrophages determines the survival of bacilli inside macrophage [8].

We investigated the probable mechanism responsible for the decline of the bacilli survival inside macrophages. Reactive nitrogen intermediates (RNIs) produced by activated mouse macrophages has been reported as one of the major element involved in antimicrobial activity [9] and mice with mutations in the gene encoding the macrophage-localized cytokine inducible nitric oxide synthase gene (iNOS) were more susceptible to *M. tuberculosis* infection [10]. Reactive nitrogen intermediates are the most significant weapon against virulent mycobacteria in mouse macrophages [11]; [12] and resistance to RNIs among various strains of *M. tuberculosis* correlates with virulence [13]. The presence of RNIs in human macrophages and their potential role in disease has been the subject of controversy, but the alveolar macrophages of a majority of TB-infected patient exhibit iNOS activity [14]. We found no significant change in concentration of nitric oxide between control, peptide and LPS treated cells. There was even no increase in the concentration of nitric oxide after infection with *M. bovis* BCG (data not shown) as compared to uninfected control cell. This suggests that RNIs are not responsible for the anti-mycobacterial activity in THP-1 cells. This is in line with the previous reports wherein it was shown that THP-1 cells, after *M. tuberculosis* infection, produce low levels of oxygen radicals and do not produce nitric oxide [15].

We studied the effect of NS and LPS on the expression of pro-inflammatory cytokine (TNF-α) and chemokines (IL8, MCP-1, MIP-1β and Rantes) at transcriptional level by RT- PCR. Pro-inflammatory cytokine and chemokines production is increased by classical macrophage activators like LPS and plays important role in making host resistant against *M. tuberculosis* infection. In our studies, we did not observe any appreciable difference at the transcripts levels of these cytokines and chemokines, while the LPS treatment induced a significant increase in the cytokines and chemokines transcripts, as reported earlier [16]. This suggests the involvement of some other mechanism in peptide mediated increased clearance of *M. bovis* BCG from THP-1 cells.

Using proteomic approach we found a Basic transcription factor, BTF3 to be significantly down regulated after treatment with NS. After western blotting transcriptionally active isoform of BTF3, BTF3a was found to be down regulated upon treatment with peptide and LPS. Peptide and LPS also caused transcriptional level down regulation of BTF3a. The BTF3 is an important transcription factor which is involved in the initiation of transcription by RNA polymerase from the proximal promoter elements such as TATA box and CAAT box sequences [17]; [18]; [19]. The BTF3 gene encodes for two proteins, BTF3a and BTF3b generated by alternating splicing. The BTF3a has the transcription characteristics of BTF3, while the BTF3b lacks the first 44 amino acids of BTF3 and is transcriptionally inactive [20]. Functional mutation of the BTF3 in mice leads to death in the early stages of development [21], pointing to an important role of this gene during normal development. In addition to its function as a transcriptional regulator, BTF3 was also found to be involved in cell cycle regulation and apoptosis [22]; [23]. For example, altered BTF3 was found to be associated with apoptosis in BL60 Burkitt lymphoma cells [24]. Down regulation of BTF3 was found to be involved in the inhibition of transcription and protein synthesis in apoptotic K562 cells [25].

Since the BTF3 was found to be down regulated in the apoptotic cells [23]; [24]; [25], the LPS mediated decrease in the BTF3a expression may be linked to the induction of apoptosis in THP-1 cell after LPS treatment. LPS has been reported to induce the apoptosis in THP-1 cell by autocrine secretion of TNF-α resulting in DNA fragmentation and caspase-3 activation [26]. BTF3 protein have putative caspase recruitment domain and caspase-3 cleavage site, and human BTF3 was found to be cleaved by caspase-3 in vitro [23]. Thus, LPS mediated caspase-3 activation may lead to degradation of BTF3 protein. NS also caused down regulation of BTF3 gene expression and BTF3 silencing was found to induce apoptosis in *C. elegans* [22], so we argued that BTF3 down regulation may augment BCG induced apoptosis in THP-1 [27]. Apoptosis induced by mycobacterium infection in host macrophage was linked with the killing of intracellular mycobacteria [28]. This suggests that programmed cell death of the host macrophage eliminates a preferred growth niche for *M. tuberculosis.* Augmentation of BCG mediated apoptosis on NS treatment may result in decreased survival of BCG inside THP-1. However pathogenic mycobacterial strain has been reported to induce little or no apoptosis above background level [29]; [30]. Thus, whether NS mediated increased BCG clearance will be able to overcome anti apoptotic action of virulent mycobacterial strains need to be further explored.

BTF3 has been found to be over expressed in cancer cells like sporadic colorectal cancer [31], glioblastoma multiforme [32] and pancreatic cancer cells [33], and it was argued that the down regulation of BTF3 gene in cancer cell may result in increased apoptosis of these cells, but this aspect is still under investigation. The confirmation of this fact will allow this peptide to be used as potential anti cancerous agent. We have thus identified BTF3 as a novel target of NS which can have a great potential in field of therapeutics.

## Materials and Methods

### Materials

RPMI1640 (Sigma), Antibiotic antimycotic solution (Sigma), Fetal bovine serum (FBS) Gibco BRL); phorbol 12-myristate 13-acetate (PMA) (Sigma Chemical Company, USA); Pure link RNA extraction kit (Invitrogen Corp., UK) and SuperScript® III Platinum® Two-Step qRT-PCR Kit with SYBR® green (Invitrogen Corp., UK), IPG strips (GE Healthcare), BTF3 antibody (abcam, UK) were purchased from the manufacturers.

### Synthesis of Peptides

Synthesis of human β -casein fragment was carried out by step wise chain elongation using solution phase method of peptide synthesis. Boc group 6 and the benzyl group were employed for the protection of a-amino and carboxyl functions respectively except Val at position 1 where Z group 7 was used for a-amino protection in order to achieve simultaneous removal of Z and benzyl group by catalytic hydrogenations in the last step of the synthesis. DCC/HOBt 9 was used as coupling reagent for preparation of peptide bond. Boc group was removed by treating the peptide derivatives with HC1/dioxane in the presence of thioanisole. Z and benzyl groups were removed by catalytic hydrogenation. The peptide was characterized by Mass spectroscopy. Homogeneity of peptide was established by TLC and reversed phase HPLC prior to use.

### Construction of Recombinant *M. bovis* BCG

The recombinant *M. bovis* BCG (Pasteur strain) carried a Fire fly luciferase gene (Flux) in fusion with *M. bovis hsp60* constitutive promoter. The fusion construct was integrated in the *M. bovis* BCG genome via an integrative plasmid vector (pMV361). The decline in bacterial growth of the recombinant strain, measured in colony forming units (CFU), corresponded with decline in relative luciferase units (RLU) (see Figure S1). RLU measurement was done using Promega Luciferase kit on Berthold (Germany) Luminometer. Recombinant *M. Bovis* BCG was grown in Sauton’s media supplemented with hygromycin (50 µg/ml) and log phase bacterial cells were taken for infection. For experimental analysis always ≥10^5–6^ bacterial cells were taken so that the error in RLU measurement remains minimal.

### Infection of THP1 Cell Line with BCG and Data Acquisition

THP-1 cells were differentiated by adding 30 nM PMA for 48 hrs in 24 well culture plates (0.2 million cells per ml per well). Differentiated THP-1 cells were treated for 24 hrs with peptides. Cells were then infected for 2.30 hrs with *M. bovis* BCG at a multiplicity of infection of 10. Following infection, non-phagocytosed bacilli were removed by washing cells thrice with RPMI 1640 medium and extracellular mycobacteria were removed by incubating cells in amikacin (200 µg/ml) containing RPMI media for 1 hour. Amikacin was removed by washing cells twice with media followed by incubation with peptides for 24 and 48 hrs. After incubation THP-1 cells were washed twice with PBS and lysed using sterile 0.05% SDS. Cell lysates were centrifuged to pellet cell debris and BCG. BCG was lysed by resuspending the pellets in 1X Passive Lysis Buffer (Promega, USA) and sonicating at 30 amplitude, 5 pulses of 3 seconds each. Luciferase activity was measured in cell lysate as described earlier. Percentage clearance was calculated after 24 and 48 hrs of infection by comparing the RLU of treated cells with respect to RLU of untreated control at each time points.

### Nitric Oxide Estimation

Concentration of nitrite produced by cells as a measure of the production of NO was determined as described earlier [34]. Briefly, 100 µl supernatant was removed from each culture well after 24 and 48 hrs of infection, centrifuged at 400 g for 10 min to make it cell free and incubated with 100 µl of Griess reagent (1% Sulphanilamide, 0.1% Napthyl ethylene diamine dihydrochloride, 2.5% Phosphoric acid) at room temperature for 10 min. Absorbance was read at 540 nm in spectrophotometer. The concentration of nitrite (NO_2_) was determined by using sodium nitrite as standard. Cell free medium was used as blank for the assay.

### Real-Time PCR

THP-1 cells were differentiated by adding 30nM PMA for 48 hrs in 24 well cell culture plates (0.2 million cells per ml per well). After 48 hrs of incubation media was changed daily for three consecutive days; on fourth day cells were treated with peptide for 6 hrs. Total RNA was extracted from treated cells using Invitrogen Purelink RNA Mini kit. After DNase treatment RNA (5 µg) was reverse transcribed using one step RT-PCR kit (Invitrogen) following manufacturès instruction. Real-Time PCR was done using tested primer set (Table. 1) and Platinum SYBR Green qPCR supermix-UDG mix (Invitrogen) following manufacturès instruction on Rochès 480 Real Time PCR Instrument. Relative quantification of cDNA was done using delta delta CT method following the normalization with Beta actin gene.

**Table 1:** List of primers used for amplification in RT PCR.

Genes	Forward primers (5′---3′)	Reverse primers (5′---3′)
TNFα	GACCTCTCTCTAATCAGCCCTCTG	CAGCCTTGG CCCTTGAAGAGGAC
IL8	CTTCCAAGCTGGCCGTGGCTCTC	TGTGTTGGCGCAGTGTGGTCCAC
Mip1beta	TCCCACCGCCTGCTGCTT TTCTTAC	GTTCAGTTCCAGGTCATACACGTAC
Rantes	CCCTCGCTGTCATCCTCATTGCTAC	TGGCACACACTTGGCGGTTCTTTC
MCP-1	CCTTCTGTGCCTGCTGCTCATAG	TCAGCACAGATCTCCTTGGCCACA
BTF3a	GGAGAGGAAGGCGATGCGACGGAC	TTCCTTTCCCACCAATGCGCACTTGTG
β actin	ATCTGGCACCACACCTTCTACAATG	ATCTTCATGAGGTAGTCAGTCAGGTC

### Sample Preparation for 2-D Gel Electrophoresis

Cells were harvested at 1500 rpm for 5 min at 4°C, and pellets were washed twice with 1X cold PBS. Traces of PBS were completely removed and cells were immediately subjected to lysis for protein extraction. 3 ml of Urea Lysis Buffer (7 M urea, 2 M thiourea, 4% (w/v) CHAPS, 1 mM EDTA, 1 mM PMSF,100 nM dithiothreitol (DTT) and protease inhibitor cocktail 10 µl/ml) was added to 10 million cells and mixed thoroughly by pipetting. Cells were lysed for one hour at RT followed by centrifugation at 14000 g for 20 min at 4°C. Supernatant was collected and ultracentrifuged at 100000 g for 1 hr at 4°C. Supernatant was collected and four equivalent volume of prechilled acetone was added and kept for precipitation at −20°C overnight. Protein was pelleted by centrifugation at 13000 g for 10 min at 4°C, supernatant was decanted and pellet was further washed with 90% prechilled acetone. After two washings pellet was air dried properly and resuspended in Rehydration Buffer (7 M urea, 2 M thiourea, 4% (w/v) CHAPS). Protein was quantified by Bradford method.

### 2-D Gel Electrophoresis

Rehydration mixture, containing approximately 150 µg of total soluble protein, was prepared in buffer containing 2% DTT, 0.5% (v/v) pH 3–10 IPG buffer in a final volume of 300 µl. Rehydration mixture was placed in rehydration tray and Immobiline Dry Strips (13 cm length, pH 3–10 linear, GE Biosciences) were placed gel side-down on top of the mixture, and the strips were allowed to rehydrate and absorb the proteins overnight at room temperature. Isoelectric focusing (IEF) was carried out on a Multiphor flat bed IEF unit (GE Biosciences) at 20°C, using a four step voltage program over 16 hrs 44 minutes with maximum voltage of 8000 V for a total of 69562 Vh. Following IEF, the IPG strips were removed from the strip holder and the cover fluid was adsorbed on filter papers. The strips were then immediately processed for second dimension SDS-PAGE gel electrophoresis. After IEF, an equilibration buffer (10 mL) was prepared that contained 0.37 M Tris-HCl, pH 8.8, 6 M urea, 20% glycerol, and 2% SDS. The buffer was divided into two parts, labeled A and B. The focused strips were removed from the Multiphor, and each strip was placed into a slotted tray, and equilibrated for 10 min with gentle rocking in 5 mL equilibration buffer A, which contained more DTT (50 mg in 5 mL) to ensure that all sulfhydryl groups were reduced. The strips were blotted, and rocked for 10 min with equilibration buffer B, which contained iodoacetamide (125 mg in 5 mL) to covalently block protein sulfhydryl groups. For the second dimension SDS-PAGE gel electrophoresis, Polyacrylamide gels (12%) were casted and the focused strips were applied to the glass plate at the top of the SDS gel cassettes. The cassettes were placed in the electrophoresis assembly (GE Biosciences). The gel was electrophoresed initially at 80 volt for 1 hour and thereafter at 120 volt till the end. To visualize the protein spots, silver staining of the polyacrylamide gels was done using Plus One Silver Staining Kit (GE Healthcare).

### Analysis of 2D Gel

The analysis of protein spots on the gels was performed with Image Master 2D Platinum software (GE Healthcare). The stained gels were scanned wet using Image Scanner II (GE Healthcare), and the images were saved as MEL and TIFF files. MEL files were opened with Image Master 2D Platinum software (GE Healthcare) and all images were cropped to same size to include only the resolving area of the gels. Each image was analyzed to identify valid spots. Errors of omission and commission were corrected manually on each gel after a careful visual inspection of the gel images magnified on the computer monitor. Three gels of treated and control group were analyzed together to found significant up regulation or down regulation of protein spots.

### Protein Sequencing

Protein spots showing differential expression pattern was picked and sent to (Proteomics International Pvt. Ltd, Nedlands, Western Australia) for doing mass spectrometry. Method followed by them in brief: Protein sample was trypsin digested and peptides extracted according to standard techniques of Bringans et. al. [35]. Peptides were analysed by electrospray ionisation mass spectrometry using the Ultimate 3000 nano HPLC system (Dionex) coupled to a 4000 Q TRAP mass spectrometer (Applied Biosystems). Tryptic peptides were loaded onto a C18 PepMap100, 3 µm (LC Packings) and separated with a linear gradient of water/acetonitrile/0.1% formic acid (v/v). Spectra were analysed to identify proteins of interest using Mascot sequence matching software (Matrix Science) with taxonomy set to human.

### Western Blotting

PMA differentiated THP-1 cells were treated with Beta casein fragment (54–59) natural sequence peptide (20 µg/ml) for 24 hours. Cells were harvested and washed twice with ice-cold PBS and sonicated on ice in lysis buffer at 10 amplitude giving 4 pulses of 5 second on and 5 second off. Lysed sample was then centrifuged at 14,000×g (4°C for 20 min) and protein concentration was determined using the Bradford protein assay. Total protein was separated by 12% SDS-polyacrylamide gel electrophoresis and was transferred to PVDF membranes (Millipore). Membranes were treated for 2 hrs at room temperature in PBS containing 5% bovine serum albumin (Sigma) and 0.1% Tween-20. Blots were then incubated for 2 hrs at room temperature with anti-BTF3 antibodies (abcam, UK) as per manufacturer’s instructions, followed by one hour incubation with peroxidase conjugated secondary antibody. Immunoreactive bands were visualised using enhanced chemiluminescence (ECL, Pierce-Thermo Scientific USA). Blots were then stripped and reprobed with an anti β-actin monoclonal antibody (Sigma) to assess the equal loading of proteins.

## Supporting Information

Figure S1 **The recombinant** ***M. bovis*** **BCG carried a Fire fly luciferase gene (Flux) in fusion with** ***M. bovis hsp60*** **constitutive promoter.** The fusion construct is integrated in the *M. bovis* BCG genome via an integrative plasmid vector (pMV361). The decline in bacterial growth of the recombinant strain, measured in Cfu, corresponded with decline in RLU. For experimental analysis always ≥10^5–6^ bacterial cells were taken so that the error in RLU measurement remains minimal. This is the communication number 8313 of CSIR-CDRI.
(TIF)
