# Supplementary figures and images for: Human Beta Casein Fragment (54–59) Modulates M. bovis BCG Survival and Basic Transcription Factor 3 (BTF3) Expression in THP-1 Cell Line
Source: PLoS One. 2012 Sep 28;7(9):e45905. doi: 10.1371/journal.pone.0045905 (PMC3461027; doi:10.1371/journal.pone.0045905)

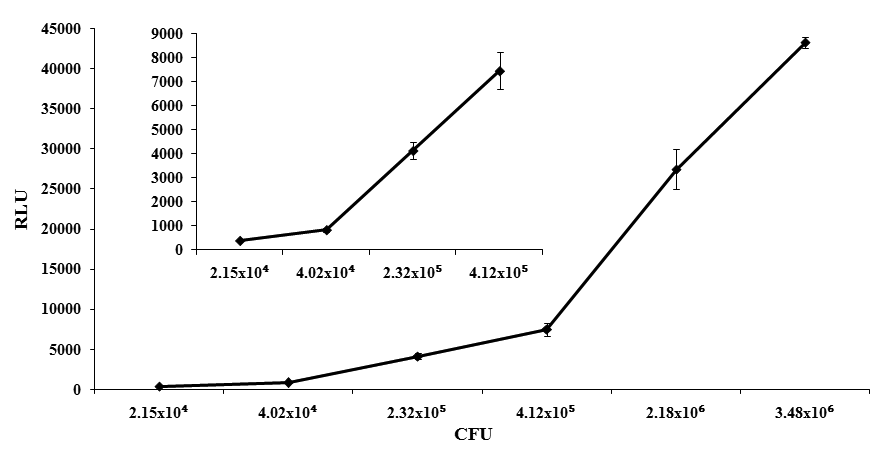

Supplement: Figure S1 — The recombinant M. bovis BCG carried a Fire fly luciferase gene (Flux) in fusion with M. bovis hsp60 constitutive promoter. The fusion construct is integrated in the M. bovis BCG genome via an integrative plasmid vector (pMV361). The decline in bacterial growth of the recombinant strain, measured in Cfu, corresponded with decline in RLU. For experimental analysis always ≥105–6 bacterial cells were taken so that the error in RLU measurement remains minimal. This is the communication number 8313 of CSIR-CDRI. (TIF) [file pone.0045905.s001.tif]
